# Splenic involvement in umbilical cord matrix-derived mesenchymal stromal cell-mediated effects following traumatic spinal cord injury

**DOI:** 10.1186/s12974-018-1243-0

**Published:** 2018-08-03

**Authors:** Anna Badner, Justin Hacker, James Hong, Mirriam Mikhail, Reaz Vawda, Michael G. Fehlings

**Affiliations:** 1 Division of Genetics and Development, Krembil Research Institute, University Health Network, 60 Leonard Ave, Toronto, Ontario M5T 2S8 Canada; 2 Institute of Medical Science, University of Toronto, 1 King’s College Circle, Toronto, Ontario M5S 1A8 Canada; 3 Division of Neurosurgery, Toronto Western Hospital, 399 Bathurst St. Suite 4WW-449, Toronto, Ontario M5T 2S8 Canada

**Keywords:** Spleen, Spinal cord injury, Mesenchymal stromal cells

## Abstract

**Background:**

The spleen plays an important role in erythrocyte turnover, adaptive immunity, antibody production, and the mobilization of monocytes/macrophages (Mφ) following tissue injury. In response to trauma, the spleen initiates production of inflammatory cytokines, which in turn recruit immune cells to the inflamed tissue, exacerbating damage. Our previous work has shown that intravenous mesenchymal stromal cell (MSC) infusion has potent immunomodulatory effects following spinal cord injury (SCI), associated with the transplanted cells homing to and persisting within the spleen. Therefore, this work aimed to characterize the relationship between the splenic inflammatory response and SCI pathophysiology, emphasizing splenic involvement in MSC-mediated effects.

**Methods:**

Using a rodent model of cervical clip-compression SCI, secondary tissue damage and functional recovery were compared between splenectomised rodents and those with a sham procedure. Subsequently, 2.5 million MSCs from the term human umbilical cord matrix cells (HUCMCs) were infused via tail vein at 1-h post-SCI and the effects were assessed in the presence or absence of a spleen.

**Results:**

Splenectomy alone had no effect on lesion volume, hemorrhage, or inflammation. There was also no significant difference between the groups in functional recovery and those in lesion morphometry. Yet, while the infusion of HUCMCs reduced spinal cord hemorrhage and increased systemic levels of IL-10 in the presence of a spleen, these effects were lost with splenectomy. Further, HUCMC infusion was shown to alter the expression levels of splenic cytokines, suggesting that the spleen is an important target and site of MSC effects.

**Conclusions:**

Our results provide a link between MSC function and splenic inflammation, a finding that can help tailor the cells/transplantation approach to enhance therapeutic efficacy.

**Electronic supplementary material:**

The online version of this article (10.1186/s12974-018-1243-0) contains supplementary material, which is available to authorized users.

## Background

Even with recent strides in patient care, spinal cord injury (SCI) remains a devastating and debilitating condition, where motor and sensory impairments hamper patient quality of life and present a major economic burden. While there is a significant need for better treatment options, few therapies succeed in clinical translation [1]. For this reason, greater understanding of injury pathophysiology, especially in relation to treatment application, will be imperative for progress to occur.

Traumatic SCI, initiated by mechanical insult known as the primary injury, involves a secondary cascade of further damage. Specifically, the disruption of local vasculature results in hemorrhage, tissue ischemia, and permeability of the blood spinal cord barrier, thereby allowing for the infiltration of peripheral immune cells and release of inflammatory cytokines [2]. Through a positive feedback loop, chemoattractants and activated immune cells further contribute to a pro-inflammatory state, which exacerbates cell death and increases tissue damage. Yet, beyond localized spinal cord changes, increasing attention has been placed on the contribution of peripheral immune organs in SCI pathophysiology [3], with considerable focus on the spleen.

Briefly, the spleen plays an important role in erythrocyte turnover, adaptive immunity, antibody production, and, more recently revealed, the mobilization of monocytes/macrophages (Mφ) following tissue injury [4]. In the context of high-level SCI, several studies have evaluated dysfunctional antibody synthesis following loss of splenic sympathetic preganglionic innervation [5–7] and the production of autoantibodies against spinal cord tissue [8]. Yet, equally important is the acute response to trauma-induced inflammation, where the spleen initiates production of tumor necrotic factor-α (TNFα), mostly by resident Mφs, and in turn influences the recruitment of immune cells to the inflamed tissue [9]. Relatedly, acute splenic atrophy and changes in the activation of residing Mφs as well as T cells have been reported in models of acute brain ischemia [10–12], as well as SCI [13]. Further, targeting this peripheral inflammatory response, via splenectomy, has been shown to significantly reduce the number of infiltrating blood-derived Mφs, thereby improving functional recovery following thoracic SCI [14]. In contrast, despite promising effects, splenectomy studies in brain injury have had mixed results [15, 16].

Notably, the application of mesenchymal stromal cells (MSCs) has further highlighted a link between the spleen and central nervous system injury. Intravenously infused MSCs have been shown to localize in the spleen following stroke [17, 18], traumatic brain injury [19, 20], and SCI [21, 22]. Although this distribution may vary with source and recipient age [23], these studies have reported dramatic changes in splenic gene expression [21], cytokine production [18, 22], and the proportion of CD4+ T cells [20] following MSC infusion. Therefore, expanding on these results, this study aimed to characterize the acute splenic cytokine response to SCI and determine its role in MSC-mediated recovery. Here, we demonstrate that cervical SCI induces a unique splenic inflammatory response, with significantly greater expression of TNFα, as early as 1 h following trauma, which is followed by a pronounced influx of other pro-inflammatory cytokines, including IL-17, IL-13, IL-1 β, and CXCL10. Despite these implications, there was no difference detected in secondary tissue damage or long-term functional recovery when compared between splenectomised rodents and those with a sham procedure. However, while the infusion of term human umbilical cord matrix (HUCMCs) reduced spinal cord hemorrhage and increased systemic levels of interleukin*-*10 (IL-10) in the presence of a spleen, these effects were lost with splenectomy. Further, the HUCMCs were also shown to alter splenic cytokine expression levels following SCI, suggesting that the spleen is an important target and site of MSC effects (when systemically infused). Taken together, these results highlight splenic involvement in acute pathophysiology and demonstrate that peripheral immune tissue may be a minimally invasive therapeutic target for SCI, with important clinical implications.

## Methods

### Clip-compression spinal cord injury (SCI)

All animal experiments were approved by the animal care committee at the University Health Network (Toronto, Ontario, Canada) in compliance with the Canadian Council on Animal Care. Adult female (approximately 250 g; 10–11 weeks old) Wistar rats (Charles River Laboratories, Wilmington, MA) were given buprenorphine (0.05 mg/kg) and 5 mL of saline prior to surgery. All rats were anesthetized with isoflurane (delivered in a 1:1 mixture of O_2_/N_2_O) and received a C7-T1 laminectomy. Next, a 35-g aneurysm clip was applied to the C7 level (1 min), modeling traumatic cervical SCI. Buprenorphine, amoxicillin trihydrate/clavulanate potassium, and subcutaneous saline injections (5 mL) were administered postoperatively. Animals were housed at 26 °C in a 12-h light/dark cycle, and their bladders were manually expressed twice a day.

### Cytokine array for splenic response to SCI & MSC infusion

The spleens of naive, laminectomy-only, and SCI animals were collected at 1 h as well as 24 h following surgery. The tissue was snap-frozen on dry ice after transcardial perfusion (with 250 ml of phosphate buffer solution; PBS) and stored for later use. When all samples were collected, the spleens were homogenized using radioimmunoprecipitation assay (RIPA) buffer (20-188, EMD Millipore) with proteinase inhibitors (Thermo Fisher Scientific), according to the manufacturer’s instructions. Total protein levels were determined using a BCA Protein Assay Kit (Thermo Fisher Scientific). The homogenate was diluted, and 100 μg of protein assessed with the cytokine R&D ELISA Proteome Profiler array (ARY008, R&D Systems Inc.) as per manufacturer’s instructions. To increase detection sensitivity, IRDye 800CW Streptavidin (926-32230, LI-COR) was used at a 1:2000 dilution (30 min, room temperature) as replacement for the kit’s streptavidin-horseradish peroxidase. The array membranes were scanned on an Odyssey Imager CLx (LI-COR), and images quantified as previously described with a semi-automated ImageJ macro [22]. The same approach was applied for the splenic cytokine profile following MSC infusion.

### Splenectomy

All Wistar rats received amoxicillin trihydrate/clavulanate potassium in water prior to the procedure. In a clean surgical space and under isoflurane anesthesia, the abdomen was accessed through a midline laparotomy. The spleen was pulled through the incision, and splenic vessels ligated prior to removal. In sham splenectomies, the spleen was returned to the abdominal cavity and the incision was closed via muscle and skin layers. Buprenorphine (0.05 mg/kg) and 5 mL of saline were administered postoperatively.

### In vivo very high-resolution ultrasound and Power Doppler imaging

In vivo very high-resolution ultrasound (VHRUS) and Power Doppler imaging was performed as previously described [24]. Briefly, a customized stabilization frame was used to fix the animal position on an imaging platform (Vevo Imaging Station, VisualSonics, Toronto, Ontario). The injury was exposed and ultrasound gel (scanning gel, Medi-Inn, Cameron, Ontario) placed on the dura mater. The VHRUS probe (44 MHz, Vevo 770, VisualSonics, Toronto, Ontario) was used to scan the spinal cord in three-dimensional (3D) B-Mode. The acute lesion and 8-week cavity volume were assessed with the TrakEM2 plugin on ImageJ software [22].

### Drabkin’s assay for intraparenchymal hemorrhage

As previously described [25], intraparenchymal hemorrhage post-SCI was assessed with Drabkin’s assay. In short, a 5-mm segment of the injured spinal cord was collected after transcardial perfusion with 250 mL of PBS. The samples were subsequently homogenized in 100 μl of distilled water and spun down at 13000 rpm for 15 min. The supernatant was collected and 20 μl added to 80 μl of Drabkin’s reagent (Drabkin’s Reagent powder [D5941] Sigma-Aldrich, in 1000 ml of distilled H_2_O and 0.5 ml of 30% Brij 35 Solution) in a clear 96-well plate. The reaction was incubated for 15 min and absorbance read at 560 nm (Wallac 1420 VICTOR2; PerkinElmer).

### Long-term neurobehavioral assessment

All neurobehavioral assessments were performed weekly (for 8 weeks) following SCI by two independent observers blinded to the experimental groups (*n* = 10 per group). As previously described, forelimb motor function was measured with a grip strength meter (SDI Grip Strength System, model DFM-10; San Diego Instruments, San Diego, CA) [26], trunk stability was evaluated with the inclined plane test [27], and hind limb locomotion was assessed by using the 22-point (0–21) Basso, Beattie, and Bresnahan (BBB) locomotor rating scale [28]. When animals reached consistent weight bearing ability (week 5–6), the CatWalk multivariate system (Noldus, Version 7.1) was used to determine detailed parameters of paw and limb function during locomotion. No food restriction or reward was used to motivate the animals to walk, and successful performance required three complete crossings with at least three consecutive step cycles for analysis. Prints were labeled by an observer blinded to the experimental groups (at week 6 and 8 post-SCI).

### Lesion morphometry

Under isoflurane anesthesia, animals were transcardially perfused with 180 mL of PBS and another 60 mL of chilled 4% paraformaldehyde (PFA). A 1-cm-length spinal cord segment (spanning the lesion epicenter) was isolated, postfixed for 1 day, and then cryoprotected in 30% sucrose before embedding in OCT medium (Thermo Fisher Scientific, Oakwood Village, OH). The spinal cord was cut into 30-μm transverse sections and stained with luxol fast blue (LFB) and hematoxylin-eosin (H&E) as previously described [29]. Lesional tissue, gray matter, and white matter percent area (sampled at every 240 μm over a distance of 2400 μm) were quantified by an observer blinded to the experimental groups using the Cavalieri probe on Stereo Investigator (MBF Bioscience, Williston, VT).

### Cell isolation, culture, immunophenotypic antigen profiling, and intravenous delivery

Umbilical cord tissue was obtained postpartum (Chelsea and Westminster Hospital, London, UK), and the veins/arteries removed under aseptic conditions. Subsequently, the Wharton’s jelly (also known as umbilical cord matrix) was diced, incubated with collagenases I and II (1 mg/ml, Thermo Fisher Scientific, Oakwood Village, OH) for 2 h (at 37 °C), and centrifuged at 2000 rpm for 10 min. The obtained human umbilical cord matrix cells (HUCMCs) were plated on culture T175 flasks (Greiner Bio-One, Monroe, NC) in minimum essential medium Eagle α modification (α-MEM) with 10% fetal bovine serum (FBS) (Wisent Bioproducts, St. Bruno, Quebec) and 0.1% gentamycin (Sigma-Aldrich, St. Louis, MO). HUCMCs were grown to 80% confluence and used at passages 7–8. Prior to in vivo application, immunophenotypic cell antigen profiling was completed with a mesenchymal cell characterization kit (1:500; SCR067, EMD Millipore, Billerica, MA) in addition to mouse anti-human CD13 (1:500; C8589, Sigma-Aldrich) and 4′,6-diamidino-2-phenylindole (DAPI; 1:1000) as nuclear counterstain. HUCMCs expressed mesenchymal cell markers sCD73, CD146, CD44, CD13, and PDGFR (Fig. 5b). For in vivo experiments, 2.5 million passage-matched (7–8) HUCMCs in 1 ml of Hanks’ buffer/2 mM EDTA (HE) were slowly infused (200 μl/minute) via the tail vein at 1 h following SCI.

### Interleukin-10 (IL-10) enzyme-linked immunosorbent assay (plasma)

Plasma levels of IL-10 were evaluated at 24 h following SCI and in SCI with HUCMC infusion. Blood samples were collected via cardiac puncture, prior to transcardial perfusion, into EDTA-coated Vacutainer tubes (K2 EDTA Plus Blood Collection Tubes, BD Biosciences, San Jose, CA). The samples were subsequently centrifuged at 3000 rpm (centrifuge 5810R, Eppendorf) for 10 min (4 °C), and the plasma was collected. The plasma samples were applied for the rat interleukin-10 (IL-10) enzyme-linked immunosorbent assay (ELISA) kit (AB100765, Abcam) per the manufacturer’s instructions, and the absorbance was read at 450 nm (Wallac 1420 VICTOR2; PerkinElmer, Waltham, MA).

### Statistical analysis

Quantitative data are expressed as the mean ± standard error of the mean (SEM). The R&D ELISA Proteome Profiler array results were normalized to laminectomy-only surgeries assessed by multiple *t* tests with Holm-Sidak correction for multiple comparisons. Other differences between groups were assessed by unpaired *t* test as well as two-way analysis of variance (ANOVA) with Tukey’s post hoc tests (statistically significant at *p* ≤ 0.05). All data were analyzed with GraphPad Prism (GraphPad Software Inc., La Jolla, CA, USA, http://www.graphpad.com).

## Results

### The spleen is a site of a SCI-induced inflammation

**Fig. 1:**
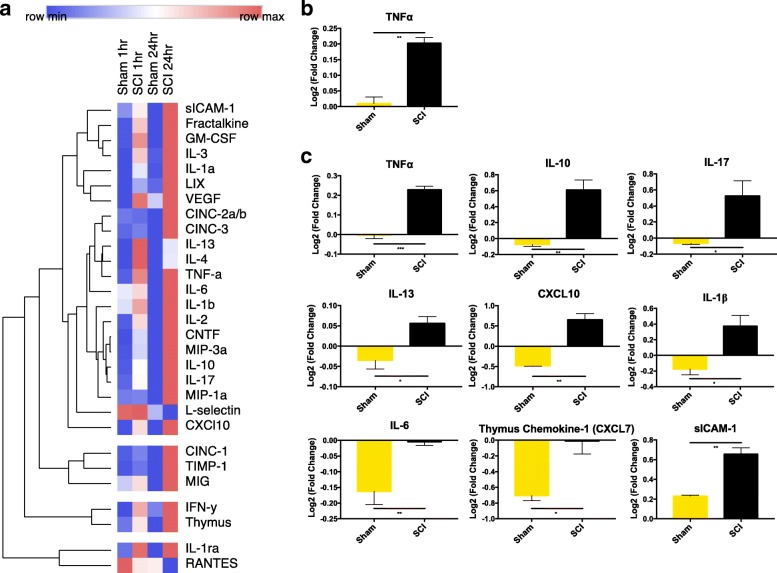
The spleen is a site of cervical SCI-induced inflammation within hours of trauma. Splenic tissue was collected at 1 and 24 h following cervical SCI and laminectomy-only surgeries. Cytokines were assessed using the rat cytokine R&D ELISA Proteome Profiler array (ARY008), and a representative heat map was generated using the BROAD Institute’s R implementation of Morpheus with Euclidean distance hierarchical clustering (**a**). Data are expressed as mean Log2 (fold change) relative to aged-matched naive spleen tissue (*n* = 4 per group). Statistically significant differences in cytokine expression (multiple *t* tests with Holm-Sidak correction for multiple comparisons) are shown at 1 h (**b**) and 24 h (**c**) following SCI between time-matched laminectomy-only surgery and injured groups

Splenic cytokine changes were evaluated with the R&D ELISA Proteome Profiler array (Fig. 1). Within the first hour following SCI, a significant (*p* = 0.002) rise in splenic TNFα levels was detected when compared to laminectomy-only surgery animals (*n* = 4 per group, multiple *t* tests, Holm-Sidak correction for multiple comparisons). This was followed by a pronounced influx in the expression of IL-17 (*p* = 0.04), IL-13 (*p* = 0.02), IL-1β (*p* = 0.03), CXCL10 (*p* = 0.002), and sICAM (*p* = 0.003) at 24-h post-SCI. Similarly, although the levels of IL-6 and thymus chemokine/CXCL7 were relatively lower than those in time-matched naive animals, expression also increased 24 h following injury. There was no significant difference in spleen weight or length (Additional file 1: Figure S1).

### Splenectomy does not reduce parenchymal hemorrhage and lesion volume following SCI

**Fig. 2:**
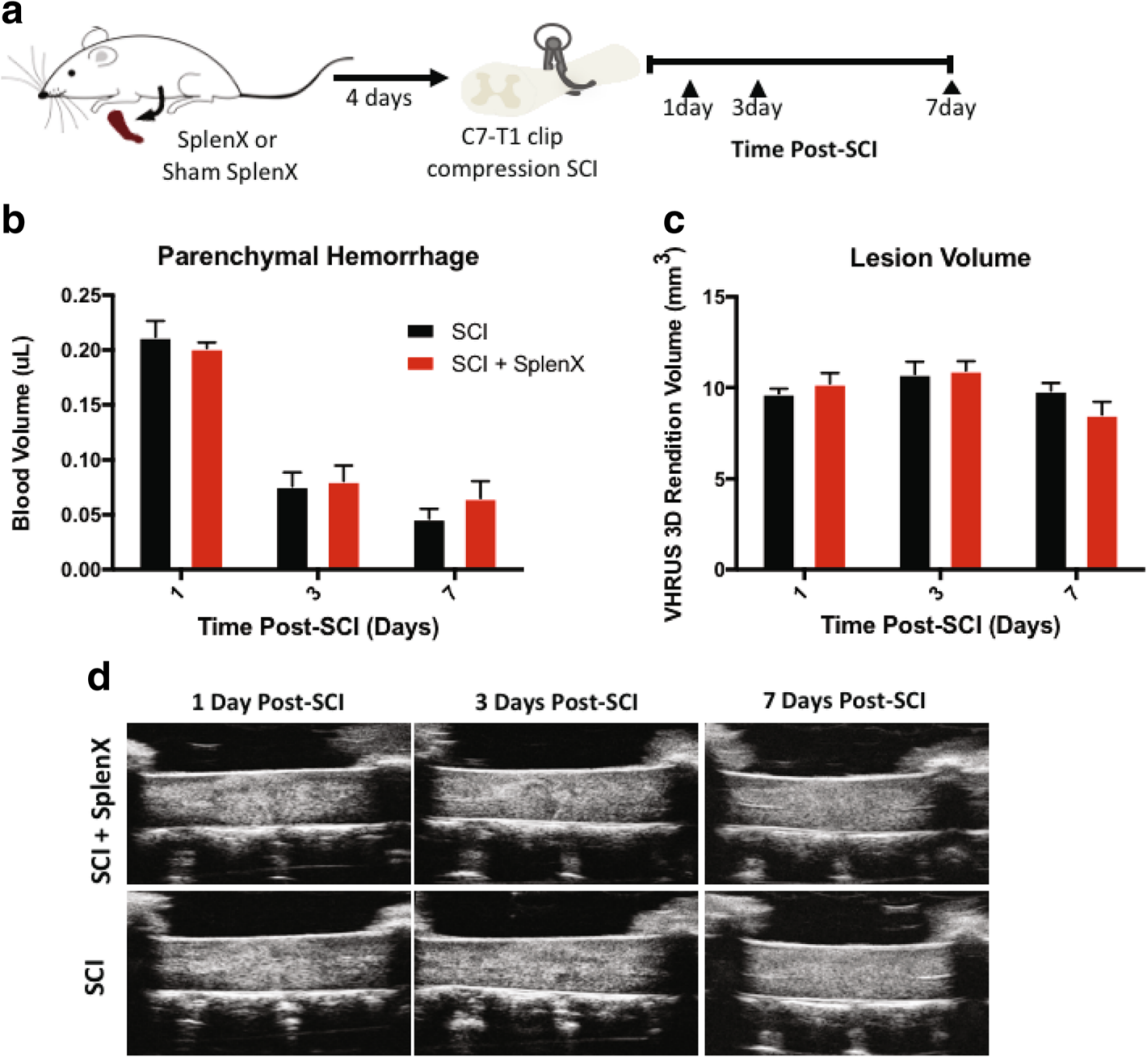
Acute spinal cord parenchymal hemorrhage and lesion volume is unaffected by splenectomy at 1, 3, or 7 days following SCI. Schematic of experimental design for acute assessments (**a**). In short, splenectomies were performed 4 days prior to clip-compression C7-T1 SCI in Wistar rats. Drabkin’s assay was applied to quantify parenchymal hemorrhage at 1 (*n* = 5 per group), 3 (*n* = 7 per group), and 7 (*n* = 5 per group) days post-SCI between the animals with or without a spleen (**b**). Very high-resolution ultrasound (VHRUS) imaging in B mode was also applied to quantify lesion volume (**c**). The representative sagittal view VHRUS images are shown at 1, 3, and 7 days post-SCI (**d**). Data are expressed as mean ± SEM, and unpaired *t* tests were performed at each time point with no significant differences found

In order to understand how the spleen contributes to secondary pathology, parenchymal hemorrhage and lesion volume were compared between splenectomised rodents versus those with a sham procedure (Fig. 2). Although SCI prompted splenic inflammation, there was no significant difference in spinal cord blood volume at 1 (*p* = 0.71), 3 (*p* = 0.25), and 7 (*p* = 0.36) days post-injury (unpaired *t* tests). Similarly, the lesion volume, quantified via in vivo VHRUS imaging, was also unaffected by splenectomy at 1 (*p* = 0.34), 3 (*p* = 0.82), and 7 (*p* = 0.07) days post-SCI.

### Splenectomy does not result in long-term functional recovery and tissue preservation following SCI

**Fig. 3:**
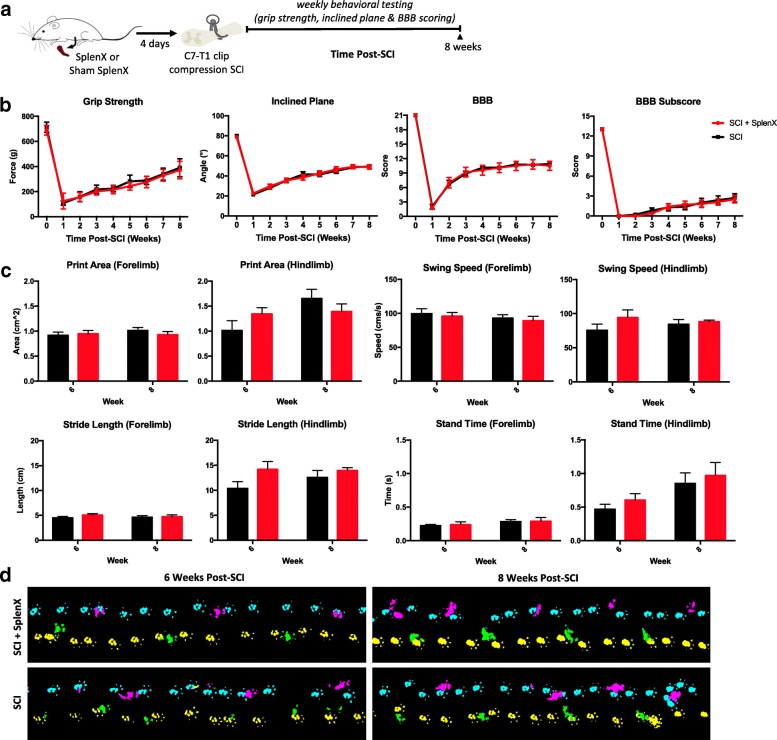
Long-term functional recovery following SCI was unaffected by splenectomy. Schematic of experimental design for long-term assessment (**a**). Briefly, splenectomies were performed 4 days prior to clip-compression C7-T1 SCI, and the Wistar rats followed for 8-weeks with weekly standard behavioral testing. There was no significant difference (*n* = 10 per group) in grip strength, inclined plane trunk stability, BBB locomotor scale, and BBB sub-score (**b**). Data are expressed as percent mean ± SEM, and two-way ANOVA analysis was performed. There was also no difference in animal gait parameters analyzed by CatWalk at 6 and 8 weeks post-SCI (**c**). Representative gait images are shown (**d**). Data are expressed as percent mean ± SEM, and unpaired *t* tests were performed at each time point with no significant differences found

Long-term functional recovery (*n* = 10 per group) was assessed with a battery of standardized neurobehavioral tests (Fig. 3). Consistent with the acute study, splenectomy did not lead to changes in grip strength (*p* = 0.07, two-way ANOVA), the inclined plane test (*p* = 0.92, two-way ANOVA), or the BBB locomotor score (*p* = 0.79, two-way ANOVA) as well as sub-score (*p* = 0.62, two-way ANOVA). Animal gait parameters were also evaluated with the CatWalk system at 6 and 8 weeks post-SCI (Fig. 3c, d). No significant differences were detected in forelimb/hind limb print area (*p* = 0.67 for forelimbs and *p* = 0.85 for hind limbs, two-way ANOVA), swing speed (*p* = 0.56 for forelimbs and *p* = 0.18 for hind limbs, two-way ANOVA), stride length (*p* = 0.28 for forelimbs and *p* = 0.07 for hind limbs, two-way ANOVA), and stand time (*p* = 0.78 for forelimbs and *p* = 0.93 for hind limbs, two-way ANOVA).

**Fig. 4:**
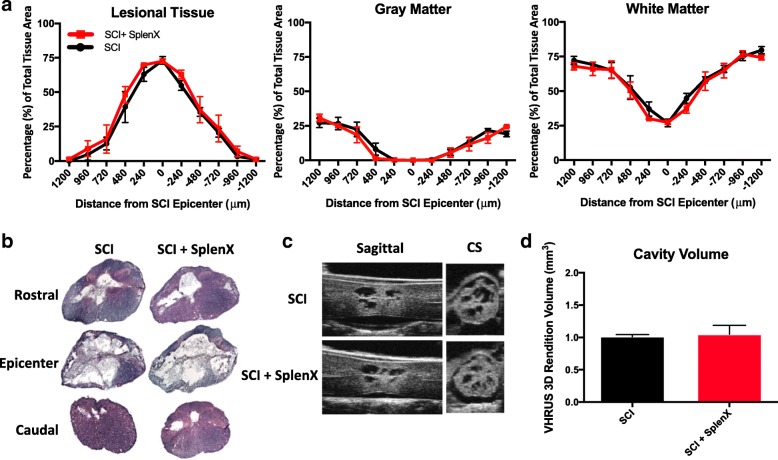
Tissue preservation and cavitation was unaffected by splenectomy at 8 weeks following SCI. Cross-sectional (30 μm) serial sections (*n* = 4 per group) were stained with Luxol fast blue and H&E and systematically sampled at every 240 μm over a distance of 2400 μm, and unbiased measurements were made with a Cavalieri probe by using Stereo Investigator. Percent area of lesional tissue, gray matter, and white matter are shown (**a**). Data are expressed as percent mean ± SEM. Two-way analysis of variance (ANOVA) with Tukey’s multiple comparison was performed, and no significant difference was found between groups. Representative images are shown rostral and caudal to the injury site, as well as at the lesion epicenter (**b**). In vivo very high-resolution ultrasound (VHRUS) imaging (*n* = 8 per group) in B mode was also used to quantify the cavity volume (**c**). Data are expressed as percent mean ± SEM, and unpaired *t* test was performed with no significant difference between groups. Representative sagittal and cross-sectional (CS) VHRUS images are shown (**d**)

The absence of changes at 8 weeks with splenectomy was further confirmed with histomorphometric analysis (*n* = 4 per group, two-way ANOVA) of LFB- and H&E-stained sections (Fig. 4). There was no effect on lesional tissue (*p* = 0.11), gray (*p* = 0.44), or white (*p* = 0.14) matter sparing. Similarly, cavity volume was unchanged (*p* = 0.78, unpaired *t* test) between splenectomised rodents (1.05 ± 0.13 mm^3^) and those with a sham procedure (1.01 ± 0.06 mm^3^).

### Splenectomy alters the acute effects of early intravenous MSCs infusion following SCI

**Fig. 5:**
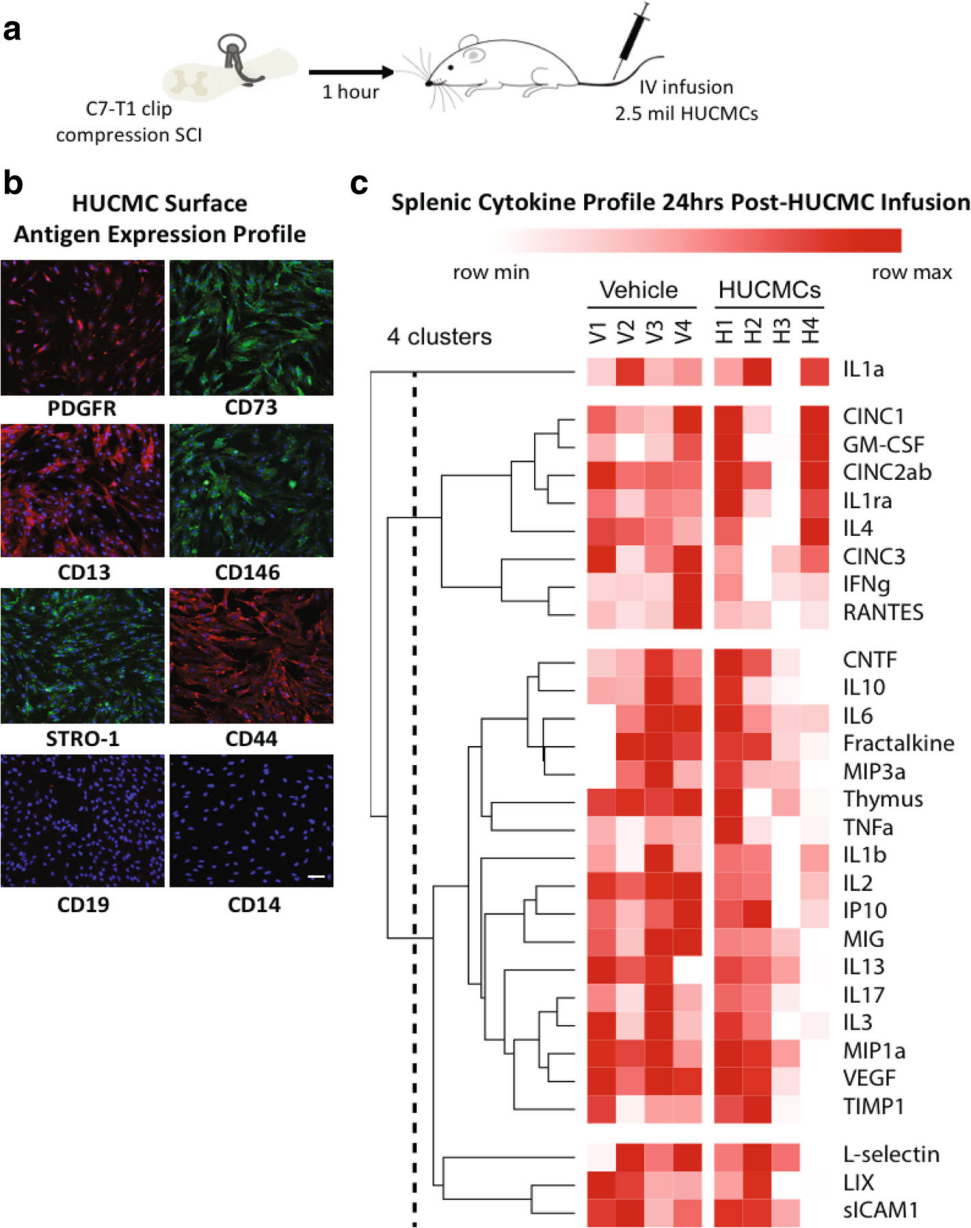
Early intravenous MSCs infusion alters splenic cytokine expression following SCI. Adult female Wistar rats (∼ 250 g) received a C7 35-g clip-compression injury and 2.5 × 10^6^ human umbilical cord matrix cells (HUCMCs) were infused 1 h following SCI (**a**). The surface antigen expression profile of MSC markers was assessed with immunocytochemistry in HUCMCs at passage 7. Representative images were taken at × 20 with the Nikon C2+ microscope (**b**). Scale bar = 10 μm. Splenic cytokines were assessed using the rat cytokine R&D ELISA Proteome Profiler array (ARY008) between vehicle and HUCMC-treated animals. A representative heat map, showing the four replicates (mean gray values), was generated using the BROAD Institute’s R implementation of Morpheus and one minus Pearson’s correlation hierarchical clustering (**c**). Statistically significant (*p* ≤ .05) differences were found for thymus chemokine/CXCL7 and sICAM (multiple *t* tests with Holm-Sidak correction for multiple comparisons)

**Fig. 6:**
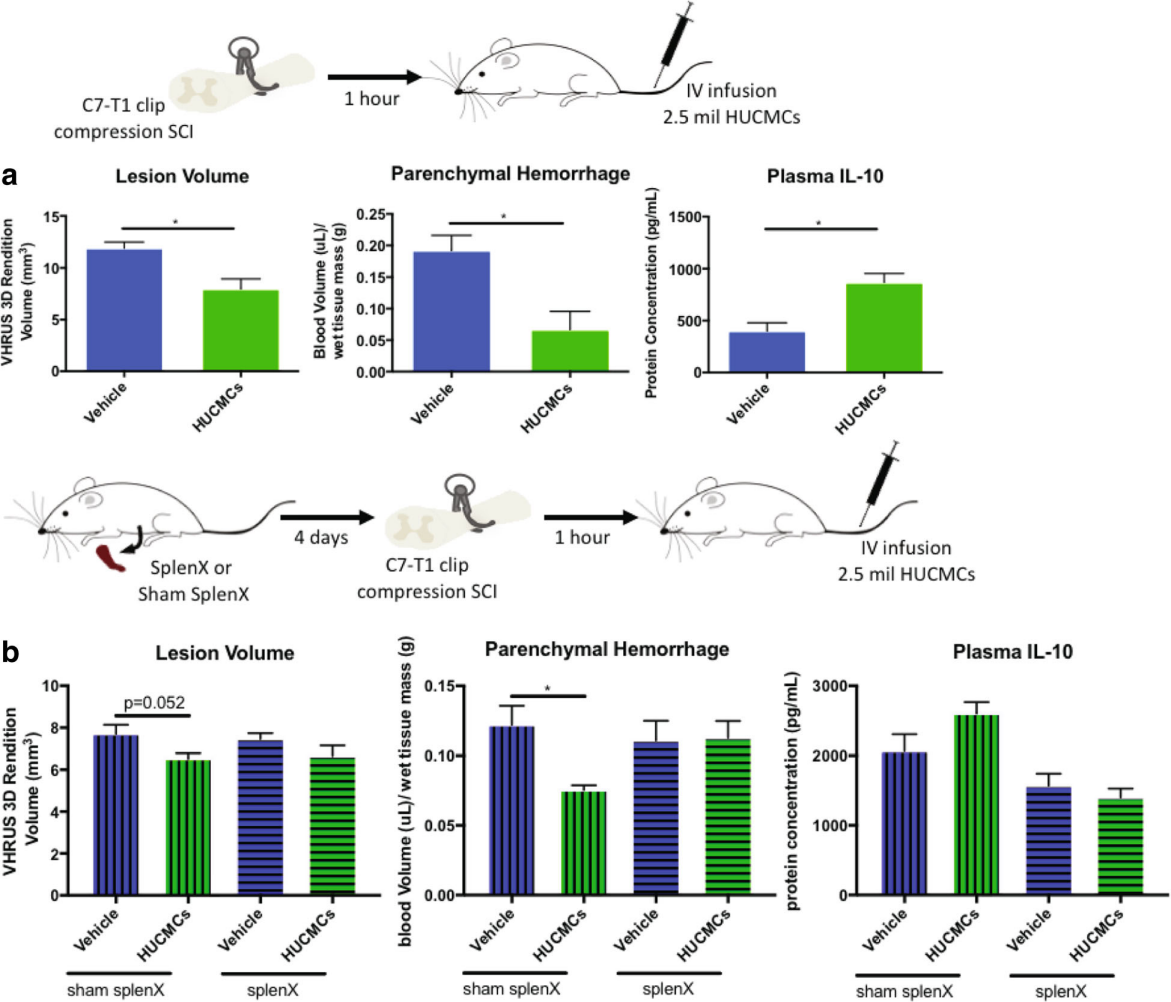
Splenectomy alters the acute effects of early intravenous MSCs infusion following SCI. When HUCMCs were intravenously infused (via tail vein) at 1 h post-SCI, there was a significant reduction in lesion volume, parenchymal hemorrhage, and plasma IL-10 at 1 day following cell transplant compared to the vehicle control (**a**). Data are expressed as percent mean ± SEM (*n* = 4 per group), unpaired *t* test, ∗*p* ≤ .05. When the experiment was repeated in the absence of a spleen (splenectomy), some of HUCMC-mediated effects at 1 day post-SCI were lost (**b**). Data are expressed as percent mean ± SEM (*n* = 4–5 per group), and unpaired *t* test performed (between HUCMCs and vehicle controls), ∗*p* ≤ .05

Prior to in vivo use, HUCMCs (passages 7–8) were validated with positive expression of mesenchymal cell markers (CD73, CD146, CD44, CD13, and PDGFR) at 80–90% confluence (Fig. 5b). Subsequently, as previously described [22], 2.5 × 10^6^ HUCMCs were infused via the tail vein at 1 h following SCI (*n* = 4 per group). The effects of cell infusion on splenic inflammation were evaluated at 1 day following SCI (Fig. 5c), where statistically significant (*p* ≤ .05) differences in cytokine expression levels were found for thymus chemokine/CXCL7 and sICAM (multiple *t* tests with Holm-Sidak correction for multiple comparisons). Spinal cord lesion volume and systemic IL-10 expression were also evaluated at 1 day post-SCI and HUCMC infusion (Fig. 6a). Animals that received HUCMCs had a significantly (*p* = 0.03, unpaired *t* test) reduced lesion volume (7.96 ± 0.93 mm^3^) compared to that of vehicle controls (11.89 ± 0.60 mm^3^). The concentration of plasma (*p* = 0.009, unpaired *t* test) and splenic IL-10 (*p* = 0.03, unpaired *t* test) was also higher in HUCMC-treated animals. Interestingly, when cells were infused to splenectomised animals, some of the effects were lost (Fig. 6b). Specifically, the HUCMC-mediated reduction in VHRUS lesion volume was absent in splenectomized animals (*p* = 0.18, unpaired *t* test), despite a trend (*p* = 0.052, unpaired *t* test) in those with a sham procedure. Similarly, while there was less parenchymal hemorrhage with cells (*p* = 0.016, unpaired *t* test) in shams, this effect was lost in the splenectomy group (*p* = 0.93, unpaired *t* test). The rise in plasma IL-10 levels was missing in both groups (*p* = 0.14 for sham and *p* = 0.44 for splenectomy, unpaired *t* tests).

## Discussion

Traumatic SCI drives local as well as systemic inflammation. Although the resulting peripheral pathology is known to contribute to multi-organ dysfunction, post-injury complications, and greater secondary damage to the spinal cord [30], the role of peripheral immune organs is poorly understood. For this reason, the therapeutic potential of lymphoid tissue targets in SCI remains largely unexplored. Here, we characterize the splenic inflammatory cytokine response to injury, demonstrating an early rise in TNFα levels that is followed by a significant influx of pro-inflammatory signaling (with increased expression of IL-17, IL-13, IL-1 β, and CXCL1). Despite previous reports [31], there was no significant difference in spleen weight or length in this injury model (Additional file 1: Figure S1). Relatedly, we further assessed the splenic involvement in secondary damage via splenectomy experiments. There was no significant difference in acute or long-term tissue damage, as well as functional recovery post-SCI, between splenectomised and sham surgery rodents. However, interestingly, when human umbilical cord matrix-derived MSCs (known as HUCMCs) were intravenously infused into splenectomised rodents, the previously reported protective effects [22] were lost. The splenic target of intravenous HUCMC infusion was further validated by demonstrating a significant reduction in inflammatory cytokines, thymus chemokine/CXCL7 and sICAM. Together, this work demonstrates that, while the inflammatory response function of the spleen can be replaced by other immune organs, it may be an important and clinically relevant therapeutic target for acute SCI pathology.

The rapid production of TNFα in the spleen post-trauma is consistent with reports in other models of injury [9], likely triggered by a combination of neural [32] as well as non-neural signaling [33], including angiotensin II. Considering TNFα’s role as a mediator of inflammation [34], the subsequent rise in splenic pro-inflammatory molecules, albeit never previously reported, was expected. These results have further implications in the context of cell therapy, where TNFα has been reported to trigger MSC immunomodulatory effects [35–37], specifically through the production of TNFα-stimulated gene-6 (TSG6). It is interesting to note that neonatal MSCs, specifically cells from the human umbilical cord, have been reported to exhibit enhanced and more rapid TSG6 expression with TNFα stimulation [38]. In turn, TSG6 has been shown to drive Mφ polarization to a more anti-inflammatory M2 state [39–41] and these cells are thought to contribute to recovery post-SCI [42, 43]. Other strategies reported to reprogram Mφs towards a more anti-inflammatory phenotype, such as peroxisome proliferator-activated receptor-*γ* (PPAR-*γ*) [44], have also shown promise in SCI [45].

It is increasingly recognized that MSCs rarely localize to the site of injury and often mediate effects through short-lived engraftment in peripheral tissue [23]. Aside from the spleen [21], as described in our previous study [22], the lungs have also been widely reported as the tissue source of MSC-mediated effects post-SCI [46] in addition to other disease models [35]. Therefore, in the absence of a spleen, one would expect the lungs to play a central role in immunomodulation with MSCs. Yet, interestingly, reduced secondary pathology (as assessed by lesion volume and parenchymal hemorrhage) as well as the systemic rise in IL-10 following MSC infusion was lost in the splenectomy animals at acute time-points. If, as suggested by the profiling data, splenic TNFα production has an important role in activating transplanted MSCs, the absence may account for the changed outcomes. However, it is important to note that other MSC effects may endure and longer time-points are needed to further understand this relationship. Relatedly, previous work with human multipotent adult stem cells derived from bone marrow, known as multipotent adult progenitor cells (MAPC), demonstrated in stroke that intravenous cell effects on long-term functional recovery were lost with splenectomy [18]. However, there was also evidence of other MAPC targets, as the effects on lesion size were preserved without the spleen.

In contrast to the aforementioned studies, the bone marrow has been traditionally recognized as the primary source of circulating Mφs [47]. While precursors are found in the spleen [48], irradiation and splenectomy studies have demonstrated that the bone marrow alone can replenish circulating populations [47]. Therefore, with bone marrow able to replace some splenic function, the unchanged acute pathology and long-term recovery in splenectomised rodents can be explained by this compensation. Nevertheless, these results differ from the work of Blomster et al., who found that splenectomy improved outcomes in mouse SCI and identified the spleen as a key reservoir of Mφs responsible for exacerbating injury [14]. These differences may stem from species variation [49] as well as timing of splenectomy relative to trauma. Future work should aim to characterize the relationship between systemic, splenic, and bone marrow immune cell population as well as phenotype changes following trauma and MSC infusion.

With considerable blood influx and a large population of immune precursor cells, targeting the spleen for therapeutic purposes has numerous advantages. In one example, systemic delivery of nanoparticles encapsulating short interfering RNA (siRNA) against CCR2 allowed for silencing expression of this chemokine in splenic populations of inflammatory Mφs, thereby attenuating their recruitment and tissue damage in various models of disease [50, 51]. Another study was also able to target the splenic Mφ progenitor recruitment by blocking angiotensin II, leading to improved outcomes in lung adenocarcinoma [52]. Together, these studies highlight the therapeutic potential of peripheral immune organ targets in neurotrauma, with the spleen as an exciting avenue for further exploration.

Herein, this work aimed to characterize a single mechanism of MSC-mediated action in the context of SCI. Although our assessment focused on the spleen, there are likely multiple mechanisms involved in the neuroprotective effects reported. For example, MSCs have been shown to coordinate the production of both matrix metalloproteinases and tissue inhibitors of matrix metalloproteinases (TIMPs) in order to mediate the structure of the vascular basement membrane and thereby vascular integrity [53]. The secretion of TIMPs (such as TIMP-3) by MSCs has been the suggested mechanism of intravenous cell delivery in a model of traumatic brain injury [19]. Other potential mechanisms include modulation of the VE-cadherin/β-catenin signaling pathway [54] and secretion of Wnt3a [55] as well as prostaglandin E2 (PGE2) [56]. Moreover, this study has been largely limited to cytokine profiling, which only provides partial insight into physiological changes. Long-term assessment and further characterization of immune cell populations is warranted.

## Conclusions

Overall, our study shows that the spleen, through amplification of inflammatory signaling, is involved in SCI secondary pathophysiology. Further, the spleen plays an important role in MSC-mediated immunomodulation, highlighting that peripheral immune tissues can be a therapeutic target for SCI. This finding can help us tailor cell therapy, as well as all systemic interventions, to maximize efficacy.

## Additional file

Additional file 1: **Figure S1.** Spleen weight and length following traumatic SCI. There was no significant difference in spleen weight (A) or length (B) at 1 or 24 h following SCI. Data are expressed as mean ± SEM (*n* = 4 per group), one-way ANOVA with Tukey’s multiple comparisons test. (PDF 354 kb)

## Abbreviations

ANOVA: Analysis of variance
BBB: Basso, Beattie, and Bresnahan
ELISA: Enzyme-linked immunosorbent assay
H&E: Hematoxylin-eosin
LFB: Luxol fast blue
MAPC: Multipotent adult progenitor cells
MSCs: Mesenchymal stromal cells
Mφ: Monocytes/macrophages
PPAR-*γ*: Peroxisome proliferator-activated receptor-*γ*
RIPA: Radioimmunoprecipitation assay
SCI: Spinal cord injury
SEM: Standard error of the mean
siRNA: Short interfering RNA
TNFα: Tumor necrotic factor-α
VHRUS: Very high-resolution ultrasound
