# Supplementary figures and images for: Splenic involvement in umbilical cord matrix-derived mesenchymal stromal cell-mediated effects following traumatic spinal cord injury
Source: J Neuroinflammation. 2018 Aug 3;15:219. doi: 10.1186/s12974-018-1243-0 (PMC6091078; doi:10.1186/s12974-018-1243-0)

**A**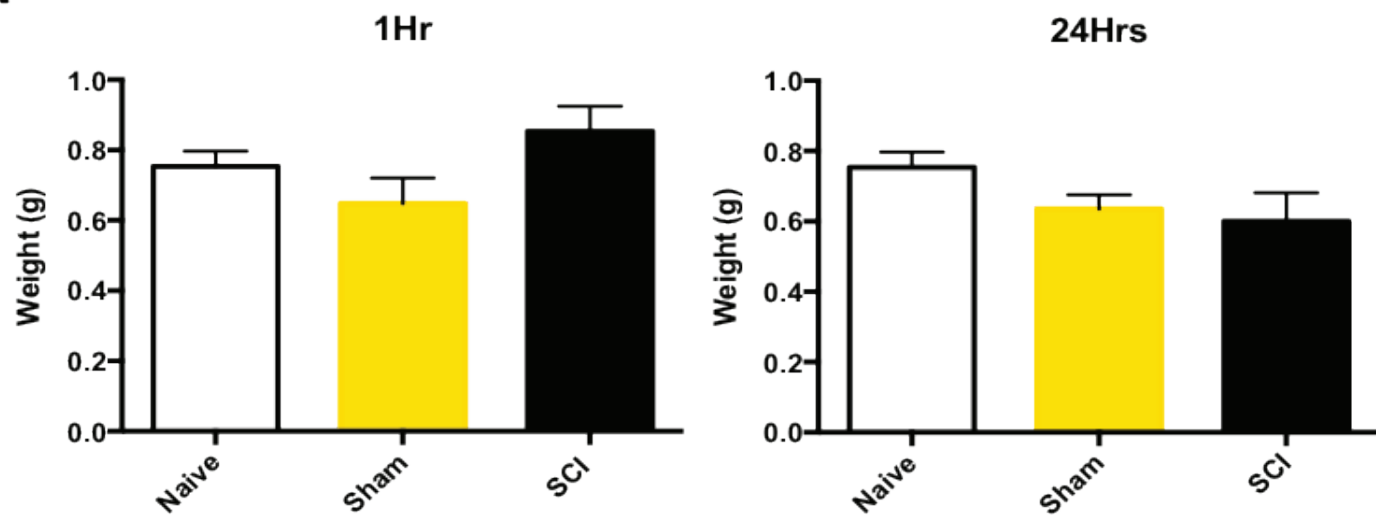**B**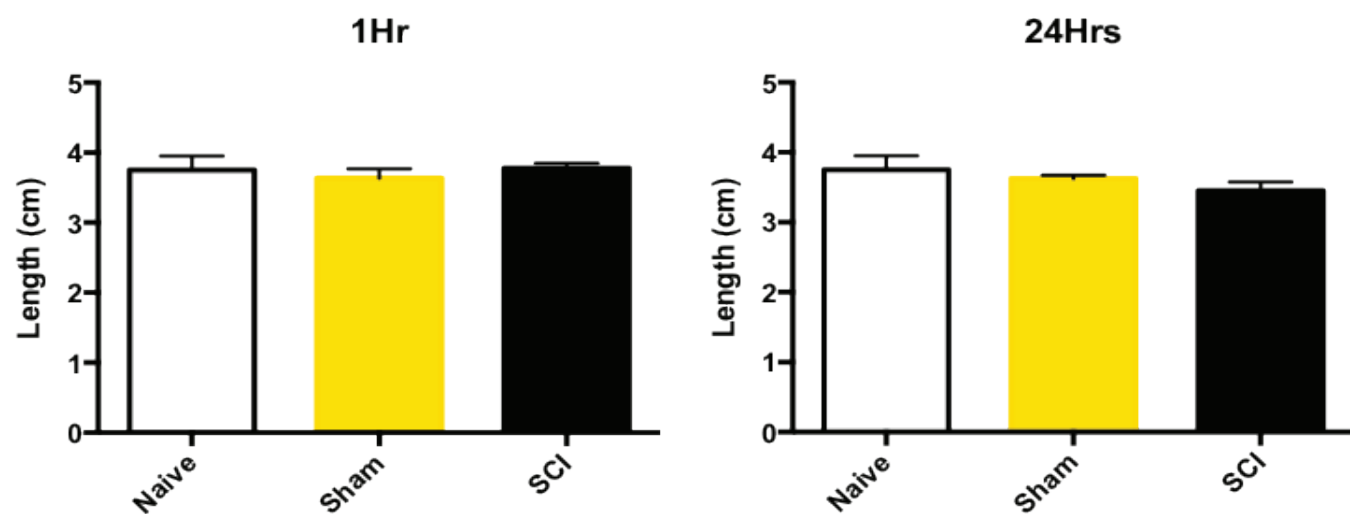

Supplement: Supplementary file 1 — Figure S1. Spleen weight and length following traumatic SCI. There was no significant difference in spleen weight (A) or length (B) at 1 or 24 h following SCI. Data are expressed as mean ± SEM (n = 4 per group), one-way ANOVA with Tukey’s multiple comparisons test. (PDF 354 kb) [file 12974_2018_1243_MOESM1_ESM.pdf]
